# Serious infection risk after 1 year between patients with rheumatoid arthritis treated with rituximab or with a second TNFi after initial TNFi failure: results from The British Society for Rheumatology Biologics Register for Rheumatoid Arthritis

**DOI:** 10.1093/rheumatology/kex304

**Published:** 2017-08-16

**Authors:** Lucía Silva-Fernández, Diederik De Cock, Mark Lunt, Audrey S Low, Kath D Watson, BSRBR-RA Contributors Group, Deborah P M Symmons, Kimme L Hyrich

**Affiliations:** 1 Arthritis Research UK Centre for Epidemiology, University of Manchester, Manchester Academic Health Science Centre, Manchester, UK; 2 Rheumatology Department, Complexo Hospitalario Universitario de Ferrol, Ferrol (A Coruña), Spain; 3 NIHR Manchester Biomedical Research Centre, Central Manchester University Hospitals NHS Foundation Trust, Manchester Academic Health Science Centre, Machester, UK

**Keywords:** Rituximab, TNF inhibitors, safety of biologics, second line biologic treatment, observational cohort, serious infections

## Abstract

**Objectives:**

Both TNF inhibitors (TNFi) and rituximab (RTX), a B-cell depleting biologic, can disrupt the immune system in RA. RTX is licensed in Europe for use following TNFi failure. However, safety data on serious infections (SIs) are scarce for RTX in daily practice. This analysis aims to compare the risk of SIs in the first year after a switch to either TNFi or RTX in patients who have failed a first TNFi.

**Methods:**

This study included patients with RA registered with the British Society for Rheumatology Biologics Register (BSRBR-RA) who switched to either a second TNFi or RTX after failing a first TNFi. Patients were followed until first SI, treatment discontinuation, last recorded follow-up or the end of the first year after the switch, whichever came first. SI was defined as requiring hospitalization, intravenous antibiotics or resulting in death. The risk of first SI was compared between TNFi and RTX using Cox proportional hazard models adjusted using propensity scores using inverse probability of treatment weighting.

**Results:**

This analysis included 3419 TNFi and 1396 RTX patients contributing 2765 and 1224 person-years (pyrs), respectively. SI occurred in 164 (4.8%) TNFi and 81 (5.8%) RTX patients giving a crude rate of 59 and 66 SI/1000 pyrs, respectively. The adjusted hazard ratio for SI was 1.0 (95% CI: 0.7, 1.4).

**Conclusion:**

The risk of SIs was comparable over the first year of treatment between TNFi and RTX treatment in patients who had failed a single prior TNFi.

**Rheumatology key messages**

- In the first year, serious infection risk is similar in patients using rituximab or a second TNFi.
- The period at risk for rituximab should be 6 months or longer after last exposure.

## Introduction

Rituximab (RTX) is a genetically engineered chimeric mAb directed against the CD20 antigen found on the surface of mature B and pre-B cells. B cell depletion therapy with RTX is an effective and relatively safe treatment for patients with RA in randomized placebo-controlled trials. RTX was licensed for the treatment of refractory RA, defined as not responding to TNF inhibitors (TNFi) therapy, in 2006 [1–3]. Treatment with RTX causes a rapid depletion of pre-B and mature B cells, which remain at low or undetectable levels for 2–6 months before returning to pre-treatment levels, generally within 12 months [4]. RTX may also cause immunosuppression through several other mechanisms such as delayed-onset cytopenia, particularly neutropenia, and hypogammaglobulinaemia, especially when administered for long periods, for example, in maintenance therapy [4]. A recent pooled observed case analysis of safety data of patients with moderate-to-severe, active RA treated with RTX in combination with MTX in a global clinical trial programme (eight randomized clinical trials and two long-term, open-label extensions) has shown that the rates of serious infections (SIs) are comparable to those observed in the placebo plus MTX population and that infection rates remain stable over time and with multiple treatment courses of RTX [5, 6]. Nevertheless, clinical trials have stringent inclusion and exclusion criteria, which make wider extrapolation of possible hazards problematic. In addition, six of the eight clinical trials included in this analysis recruited RA patients naïve to biologic therapy with TNFi [2, 3, 7–10], including one trial (IMAGE) in MTX-naïve patients [10]. In Europe, RTX is licensed only for patients who have failed initial TNFi therapy because of drug intolerance or inadequate clinical response to the drug. Hence, clinicians and patients require information on the comparative rates of SI between patients receiving RTX or a second TNFi after failing a first TNFi in order to aid treatment choice.

Only one recent trial compared head-to-head RTX with TNFi in biologic-naïve patients with RA. SI rates were low but similar between the two study arms [11]. The SI rate in 1681 patients with RA treated with RTX from the French Autoimmunity and Rituximab Registry was 50/1000 patient-years (82 episodes in 78 patients) [12]. Chronic lung disease and/or cardiac insufficiency, RA-related extra-articular manifestations and IgG level <6 g/l before the initiation of RTX were found, in multivariate analysis, to be associated with an increased risk of infections during the 12 months following a course of RTX therapy. However, there was no comparison group and RTX use at multiple points in the treatment pathway (from TNFi-naïve to fourth line use) was included.

The primary aim of our analysis was to compare the rates of SI between patients with RA treated with either RTX or TNFi who have failed to respond to a first TNFi.

## Methods

### Patient population

Patients included in this study were participants in the British Society for Rheumatology Biologics Register (BSRBR)-RA, which is a large national prospective observational study established primarily to assess the long-term safety of exposure to biologic therapies in patients with RA. Full details of the BSRBR-RA methodology have been published previously [13]. In brief, the study commenced in 2001 closely followed by national recommendations that all RA patients prescribed TNFi within the UK should be registered [14]. Patients were recruited to the TNFi cohort from 2001 onwards and all subsequent biologic therapy exposure following registration is captured, including exposure to RTX. Specific registration targeting patients at the point of starting RTX opened in 2007 to recruit patients starting this therapy who may not have already enrolled at the point of starting TNFi. Patients already in the register as TNFi patients who switched to RTX after 2007 were also eligible to be re-registered in order to capture clinical information at the point of switch (e.g. the DAS28 count [15], HAQ score [16] and patient reported safety data from the point of switching onwards. In all cases of new registration, the patient should have started RTX within 6 months prior to registration. At the time of this analysis, all patients had received Mabthera as RTX.

### Ethical approval

Ethical approval for the BSRBR-RA was granted by the North West Multicentre Research Ethics Committee in December 2000 (reference no. MREC 00/8/53). All patients provided written informed consent. No additional ethical approval was required for this study.

### Baseline assessment

Baseline information on all patients in the BSRBR-RA is collected at recruitment (or re-recruitment) and includes demographic data, disease duration, 28-joint swollen and tender joint counts, ESR and/or CRP level, patient global assessment on visual analogue scale, history of extra-articular manifestations, previous and current DMARD therapy, current steroid use and details of previous and current comorbidity from a pre-defined list. DAS28 is calculated and patients complete a HAQ [17]. For this analysis, patients who were initially registered as TNFi patients and then re-registered at point of starting RTX and for patients registering for the first time when they switched to RTX, baseline data were taken from their RTX baseline record. For all other patients, data were extracted from their original registration when starting TNFi and updated using any information obtained between start of initial TNFi and start of second TNFi (or RTX if not re-registered). We used the most recent DAS28 or HAQ measured within 6 months of starting their second TNFi.

### Follow-up

Follow-up data are captured in three independent ways: patients’ rheumatologists are sent a questionnaire 6 monthly for 3 years and annually thereafter, requesting details on changes in DMARD or biologic therapy, current disease activity and development of any adverse events including duration of any hospital admissions; patients are sent a diary 6 monthly for 3 years, requesting details on any new hospital referrals, new medication and hospital admissions; and the UK National Health Service Information Centre provides the BSRBR-RA information on the death of any of these patients, including the cause of death.

### Cohort selection

Analysis was restricted to patients with a rheumatologist’s diagnosis of RA who had failed their first TNFi for any reason and then switched to either RTX or a second alternative TNFi. Patients treated with a biologic agent other than RTX or TNFi prior to inclusion were excluded from the analysis.

The length of time in study could differ between patients switching to a second TNFi or RTX because TNFi patients are followed up from 2001 while RTX recruitment started in 2007 in the BSRBR-RA. Therefore, we restricted the analysis to those patients who started RTX or their second TNFI at registration or within 3 years following registration with the BSRBR-RA, a time period which included follow-up data collected from both the hospital and the patient. For the purpose of this analysis, baseline was defined as the date the patient initiated RTX or a second TNFI (switch-time) after having discontinued a first TNFi. The data cut-off point for follow-up data was 30 November 2015.

### Case definition and verification

SIs, defined as requiring intravenous antibiotics, hospitalization or resulting in death, were attributed to the second TNFi if they occurred while the patient was receiving TNFi or within 90 days of the first missed dose and were attributed to RTX if they occurred while the patient was receiving RTX or within the 9 months after the last infusion.

Infections were coded by anatomical site and by organism. Two methods were used to measure SI severity. First, information on the duration of hospitalization was obtained from the clinical questionnaire. Second, mortality was determined by identifying patients who died within 30 days of SI.

### Statistical analysis

All patients were followed from the switch-time (starting second TNFi or RTX) until death, first SI, last follow-up, drug discontinuation or the end of the first year after the switch, whichever came first. Patients within the TNFi cohort who stopped therapy for a reason other than SI contributed follow-up time until 90 days after their first missed dose and patients within the RTX cohort who stopped therapy for a reason other than SI contributed to follow-up time until 9 months after their last infusion. This time window was chosen as the primary risk window because B-cell levels in peripheral blood after the last RTX infusion are generally back to normal in 6–9 months [18]. In a sensitivity analysis, risk windows of 90 days, 6 and 12 months were also tested. The time to first infection in the first 12 months was visually examined using a Kaplan–Meier curve between exposure groups, with RTX patients censored if they received a second course of RTX during the 12-month period.

Crude incidence rates were calculated as the number of first episodes of SI per 1000 patient-years of follow-up with a 95% CI. Survival analyses, performed using a Cox proportional hazards model, were used to compare the rates of SIs between cohorts. To reduce the impact of treatment selection bias and potential confounding in an observational study, we used inverse probability-weighted estimates based on the probability of a patient receiving the treatment he or she actually received conditional on observed covariates [19]. We estimated these probabilities with the use of a treatment selection, or propensity, model. We fitted the propensity model as a logistic regression model with treatment as the dependent variable. The baseline covariates balanced by the propensity score model were identified from an *a priori* list including age at treatment start (second TNFi or RTX), gender, disease duration, smoking, RA disease severity, number of prior DMARDs and reason for stopping first TNFi. Additional potential confounders were identified as variables either unbalanced between the TNFi and the RTX cohorts (calendar year of starting biologic therapy, previous SI) or significant predictors of infection (lung disease, diabetes, steroid exposure).

Multiple imputation was used to replace missing baseline data. The imputation model was constructed separately for the TNFi and RTX cohorts. Age, gender, disease duration, smoking status, HAQ score, DAS28, use of steroids, reason for stopping the first TNFi, previous SI while on the first TNFi, number of prior DMARDs, co-morbidity (diabetes, lung, renal, heart and liver disease), previous cancer, tuberculosis, year of starting biologic treatment and having an SI while on RTX or a second TNFi were all included as predictors within the imputation model. Twenty data sets were imputed using the ICE package in Stata and analysed using Rubin’s rules with the MIM command. All analyses were conducted using Stata version 13 (StataCorp, College Station, TX, USA).

## Results

This analysis included 4815 patients: 3419 in the TNFi cohort and 1396 in the RTX cohort. Of the RTX cohort, 677 (48.4%) patients were recruited at point of starting RTX; the other patients were already in the register as TNFi patients switching to RTX. In the TNFi cohort, 1751 (51.2%) patients started Enbrel, 356 (10.4%) Remicade, 1191 (34.8%) Humira, 102 (3.0%) Cimzia and 19 (0.5%) Simponi as their second TNFi. The baseline characteristics of the patients are shown in Table 1. Although both TNFi and RTX treated patients were initiating their second biologic after discontinuing a first TNFi, there were significant differences at baseline. The patients receiving RTX were slightly older, they had higher disease activity and there was a higher proportion of patients with previous cancer, chronic respiratory disease and diabetes. Moreover, more patients receiving RTX as their second biologic were reported to have SIs before switching biologics. In the TNFi cohort, 6.3% (214/3419) of patients had at least one SI in the past compared with 8.8% (63/719) of patients in the RTX cohort (P = 0.015). In the RTX cohort, data on past SIs were only available for 719 patients.

**Table 1:** Baseline patients’ characteristics

Characteristic	TNFi (n = 3419)	Rituximab (n = 1396)	P-value
Age, mean (s.d.), years	55.9 (12.3)	58.3 (12.2)	0.0001
Women, n (%)	2722 (80)	1073 (77)	0.03
Disease duration, median (IQR), years	12 (6–19)	11 (5–19)	0.1
DAS28, mean (s.d.)	5.6 (1.5)	6.0 (1.2)	0.0001
HAQ score, mean (s.d.)	1.9 (0.6)	2.0 (0.6)	0.008
RF^+^, n (%)	2136 (63)	860 (67)	0.005
Steroid use, n (%)	1370 (54)	239 (62)	0.006
Lung disease, n (%)	582 (18)	259 (22)	0.002
Diabetes, n (%)	198 (6)	120 (9)	<0.001
Previous cancer, n (%)	109 (3)	101 (7)	<0.001
Smoking, n (%)			0.2
Current	768 (23)	310 (22)	
Ex	1.259 (37)	547 (39)	
Never	1385 (41)	531 (38)	
Time on first biologic before switch, mean (s.d.), years	1.0 (0.7)	1.5 (1.7)	0.0001
First TNFi therapy, n (%)			<0.001
Remicade	1310 (38)	216 (15)	
Enbrel	956 (28)	539 (39)	
Humira	1053 (31)	525 (38)	
Cimzia	98 (2.9)	114 (8)	
Simponi	2 (0.1)	2 (0.1)	
Reason for stopping first TNFi, n (%)			<0.001
Inefficacy	1846 (54)	709 (51)	
Adverse event	1080 (31)	286 (21)	
Missing	493 (14)	401 (29)	
Switched before 2007, n (%)	2443 (72)	49 (4)	<0.001

Baseline is defined as time of switching to RTX or second TNFi. TNFi: TNF inhibitors.

**Fig. 1:**
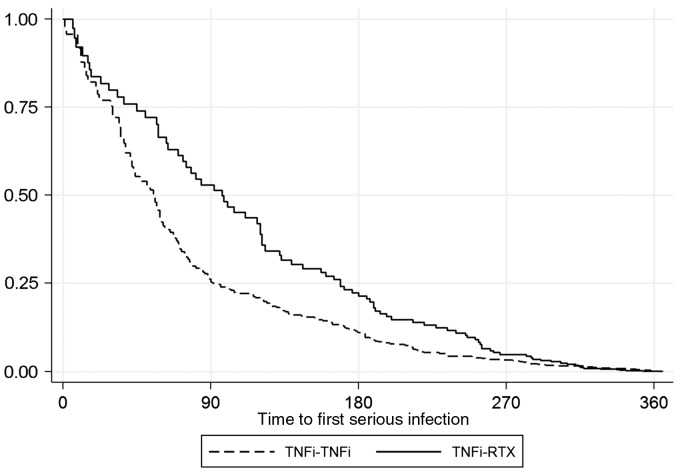
Kaplan–Meier curve showing time to first serious infection by exposure group A 90 days exposure for TNFi–TNFi was applied. A 365 days exposure window for TNFi–RTX was applied. RTX exposure time was also censored after one course of RTX. The time to first serious infection is clearly different between exposure groups after 90 days, but after 6 months this difference diminishes. This figure advocates a longer exposure window for RTX, preferably 9 or 12 months. TNFi: TNF inhibitor; RTX: rituximab.

In total, 245 patients experienced at least one SI (TNFi: 164; RTX: 81; Table 2) within the 12 months following treatment start (follow-up censored at 1 year). The crude rate of SIs was 59 events/1000 person-years (pyrs) in the TNFi cohort and 66 events/1000 pyrs in the RTX cohort, with an unadjusted hazard ratio (HR) for the RTX group of 1.1 (95% CI: 0.8, 1.4). Neither adjustment for age and sex (HR = 1.0, 95% CI: 0.8, 1.3) nor further adjustment using propensity scores (HR = 1.0, 95% CI: 0.7, 1.4) appreciably affected the HR. The median time to first infection was similar between the two treatment groups (TNFi 0.3 years; RTX 0.4 years). Following a single course of RTX, 90% of first SIs were observed by month 9 (Fig. 1) giving support to the choice of the 9-month risk window as the primary window for analysis. Sensitivity analysis using other risk windows following each course of RTX found a lower rate of infection among patients receiving RTX compared with TNFi if only infections in the first 3 months following RTX treatment were included, but there was no difference when the risk window for RTX was increased to 6 months or 12 months (Table 3). Because RTX treatment for RA was only available from 2007, fewer than 5% of the RTX cohort received the drug before 2007 whereas 72% of patients on their second TNFi had switched before this year. Therefore, we conducted a sensitivity analysis confined to patients who switched to a second biologic from 2007 onwards. The unadjusted HR for SI in patients starting RTX in comparison with those starting a second TNFi after 2006 was 1.3 (95% CI: 0.9, 1.9) and the fully adjusted HR was 1.5 (95% CI: 0.7, 3.2) (Table 2).

**Table 2:** Overall risk of serious infection

	Whole cohort		Switched after 2007	
	TNFi	Rituximab	TNFi	Rituximab
Number of patients	3419	1396	976	1347
Follow-up, pyrs	2765	1224	758	1138
Follow-up time per patient, median (IQR), years	1.0 (0.7–1.0)	1.0 (0.8–1.0)	1.0 (0.5–1.0)	1.0 (0.8–1.0)
Number of first SIs	164	81	38	75
Time to infection, median (IQR), years	0.3 (0.2–0.5)	0.4 (0.2–0.6)	0.3 (0.2–0.4)	0.4 (0.2–0.6)
Crude incidence rate of SI/1000 pyrs (95% CI)	59 (51, 69)	66 (53, 82)	50 (36, 68)	63 (51, 79)
Unadjusted HR (95% CI)	Ref.	1.1 (0.8, 1.4)	Ref.	1.3 (0.9, 1.9)
Age and gender adjusted HR (95% CI)	Ref.	1.0 (0.8, 1.3)	Ref.	1.2 (0.8, 1.8)
Fully adjusted by IPTW HR (95% CI)	Ref.	1.0 (0.7, 1.4)	Ref.	1.5 (0.7, 3.2)

HR: hazard ratio; IPTW: inverse probability of treatment weighted method; pyrs: patient-years; SI: serious infection; TNFi: TNF inhibitors.

**Table 3:** The influence of different periods at risk for Rituximab on serious infection rates

	Whole cohort	
	TNFi	Rituximab
Number of patients	3419	1396
RTX 90-day time window		
Number of first SIs	164	42
Crude incidence rate of SI/1000 pyrs (95% CI)	59 (51, 69)	38 (28, 51)
Unadjusted HR (95% CI)	Ref.	0.6 (0.4, 0.8)
RTX 6-month time window		
Number of first SIs	164	68
Crude incidence rate of SI/1000 pyrs (95% CI)	59 (51, 69)	56 (44, 72)
Unadjusted HR (95% CI)	Ref.	0.9 (0.7, 1.1)
RTX 9-month time window		
Number of first SIs	164	81
Crude incidence rate of SI/1000 pyrs (95% CI)	59 (51, 69)	66 (53, 82)
Unadjusted HR (95% CI)	Ref.	1.1 (0.8, 1.4)
RTX 1-year time window		
Number of first SIs	164	88
Crude incidence rate of SI/1000 pyrs (95% CI)	59 (51, 69)	67 (54, 82)
Unadjusted HR (95% CI)	Ref.	1.1 (0.8, 1.4)

Unadjusted risk rates of serious infections in regards to different periods at risk for Rituximab; hazard ratio for Rituximab using TNF inhibitors as a comparator. SI: serious infection; HR: hazard ratio; TNFi: TNF inhibitors; RTX: rituximab.

Table 4 shows the site of SI by treatment group. Lower respiratory tract and lung infections were most frequently reported in both groups. In the majority of cases (84.1%), no organism was identified and/or reported in the study. Where reported the majority of cases were due to non-opportunistic bacterial organisms. There were no cases of tuberculosis reported. There was one case of *Aspergillus* pneumonia and two cases of intracellular bacterial infections (one *Listeria* and one *Legionella*) among patients receiving TNFi. No participant died within 30 days following an SI. The median (IQR) duration of hospitalization following an SI was 6 (4–10) days in the RTX group and 5 (2–11) days in the TNFi group (P = 0.5).

**Table 4:** Site of first serious infection

Type of infection (MedDRA HLT)	TNFi, n (%)	Rituximab, n (%)
Lower respiratory tract and lung infections	62 (37.8)	38 (46.9)
Urinary tract infections	23 (14)	8 (9.9)
Bacterial infections NEC	18 (11)	5 (6.2)
Bone and joint infections	17 (10.4)	5 (6.2)
Skin structures and soft tissue infections	14 (8.5)	8 (9.9)
Abdominal and gastrointestinal infection	8 (4.9)	2 (2.5)
Hepatobiliary and spleen infections	6 (3.7)	2 (2.5)
Dental and oral soft tissue infections	3 (1.8)	1 (1.2)
Infections NEC	4 (2.4)	4 (4.9)
Upper respiratory tract infections	3 (1.8)	0
CNS and spinal infections	2 (1.2)	1 (1.2)
Sepsis, bacteraemia, viraemia and fungal	2 (1.22)	5 (6.2)
Male reproductive tract infections	1 (0.6)	0
Muscle and soft tissue infections	1 (0.6)	0
Eye and eyelid infections	0	1 (1.2)
Influenza viral infections	0	1 (1.2)
**Total**	**164 (100)**	**81 (100)**

MedDRA HLT: Medical Dictionary for Regulatory Activities High Level Term; n: number of infections; NEC: not elsewhere classified; TNFi: TNF inhibitors.

## Discussion

In this study, we demonstrated that the risk of SI in the first year is similar between patients receiving RTX and those receiving a second TNFi after failure of a first TNFi in a large observational cohort. The most common types of SI in both groups were lower respiratory tract infections followed by urinary infections, consistent with results of previous studies [1, 3].

The influence of RTX therapy on infection risk has primarily been studied in patients with haematological malignancies. Adding RTX to standard chemotherapy is shown to increase the risk of severe leukopenia and granulocytopenia during therapy [20]. Moreover, the use of RTX as a maintenance therapy for lymphoma increases the infection rate compared with placebo [21]. The influence of RTX therapy on infection risk is complex in the context of RA. Firstly, different RTX dosing regimens are used in RA practice [22]. Secondly, RA patients receiving RTX have often received multiple and varied previous treatments, including synthetic DMARDs, biologic DMARDs and glucocorticoid therapy [22]. Additionally, the increased baseline risk of infections in patients with RA makes the attribution of adverse events to RTX even more complex [23]. Narrative reviews of published articles ([4, 24] have not found an increased risk of infections in various RA populations receiving RTX. The most recent data from the Rheumatoid Arthritis Global Clinical Trial Program [6], an analysis of pooled data from 3595 participants in randomized clinical trials who received a mean of four courses of RTX over 11 years (14 816 pyr), has shown a similar rate of SI of 3.76 (95% CI: 3.46, 4.09) per 100 pyr in patients exposed to RTX compared with 3.79 (95% CI: 2.80, 5.13) per 100 pyr in a pooled placebo population of 818 RA patients. Unfortunately, this population does not reflect RTX use in routine clinical practice, as a large majority of the patients included had not received a TNFi previous to RTX and some patients were even MTX-naïve [5]. A recent large observational study by Aaltonen *et al.* [25] showed no difference in infection rates in patients treated with TNFi, RTX or conventional DMARD therapies, yet this study pooled all RTX patients together regardless of the context in which RTX was prescribed.

A first strength of our study is that the majority of patients received the standard RTX dose of twice 1000 mg in an interval of 14 days, as it was approved for and the recommended dose during the period of the study. Another major strength of our study is that our patient population is strictly defined and homogeneous [13]. The risk of SI is compared between patients receiving RTX or a second TNFi after failing a first TNFi, the two most common scenarios in daily practice in the UK [26]. Prior to RTX approval for RA in 2007, the use of a second TNFi was the only biologic treatment option for RA patients in the UK [27]. It is still common in Europe [28] and the approach is recommended when RTX is contraindicated [26]. An additional strength is the systematic follow-up in the BSRBR-RA register, limiting missing data and designed to capture most AEs as close to occurrence as possible, and thus allowing for a reliable estimation of biologic treatment safety.

When studying the risk of infection following RTX, it is unknown for what period of time patients remain at risk of infection or how long a risk window to include in analyses. In our study, by 6 months, 75% of patients who would experience an infection in the first year after RTX had already done so, but a further 15% experienced an infection in the following 3 months, suggesting that in some patients this risk of infection continues beyond 6 months. In this study, a risk window of 9 months was chosen as the primary analysis, but we found that no difference in risk between TNFi and RTX was observed after 6 months. The risk of infection was higher in patients receiving TNFi during the first 3 months, which is consistent with prior reports of an early risk of infection following TNFi [29, 30].

Weaknesses include the fact that this is an observational study and so is limited due to potential confounding because of non-random assignment of subjects to treatment. This channeling bias is underlined by the baseline differences between the two treatment groups. We tried to minimize this bias by adjusting for multiple covariates that may potentially confound any association between biologics and SI using propensity scores. Because RTX use was not approved before 2006, our study comprised a period of 5 years during which only one treatment option (a second TNFi) was available. However, in a sensitivity analysis confined to patients switching biologics after 2006, no differences were found in the rates of SI. Another limitation is that we have only investigated the infection rate in the first year. Randomized clinical trials as well as observational cohort studies have shown an increased risk of SI at the start of TNFi treatment, particularly in the first 6 months of treatment [29]. However, this risk decreases over time to reach the same level as conventional therapies after 1 year of treatment [31]. Therefore, this analysis cannot comment on the risk of infections in the long term and with repeated RTX infusions. Another important limitation is the absence of information on steroid dosing in the BSRBR-RA register. Steroid dosing is known to influence infection risk, but unfortunately we cannot adjust for it in our analysis.

In conclusion, our study found no difference in the risk of SI over the first year of treatment in patients treated with RTX compared with those treated with a second TNFi after discontinuing a first TNFi. This information should be of value to clinicians and patients when choosing a second biologic in RA.
